# Integrated Machine Learning and Single-Sample Gene Set Enrichment Analysis Identifies a TGF-Beta Signaling Pathway Derived Score in Headneck Squamous Cell Carcinoma

**DOI:** 10.1155/2022/3140263

**Published:** 2022-09-01

**Authors:** Sheng Wu, Xiangkang Lv, Yilin Li, Xinyi Gao, Zhiqi Ma, Xiao Fu, Yong Li

**Affiliations:** ^1^The Fourth School of Clinical Medicine, Zhejiang Chinese Medical University, Hangzhou, Zhejiang 310053, China; ^2^The Second School of Clinical Medicine, Zhejiang Chinese Medical University, Hangzhou, Zhejiang 310053, China; ^3^Department of Otolaryngology Head and Neck Surgery, Affiliated Hangzhou First People's Hospital, Zhejiang University School of Medicine, Hangzhou, Zhejiang 310000, China

## Abstract

**Background:**

The TGF-*β* signaling pathway is clinically predictive of pan-cancer. Nevertheless, its clinical prognosis and regulation of immune microenvironment (TME) characteristics as well as the prediction of immunotherapy efficacy need to be further elucidated in head and neck squamous cell carcinoma.

**Method:**

At first, we summarized TGF-*β* related genes from previous published articles, used ssGSEA to establish the TGF-*β* risk score. Considering the complexity of its clinical application, we improved it with the LASSO-COX algorithm to construct the model. In addition, we explored the predictive efficacy of TGF-*β* risk score in the observation of TME phenotype and immunotherapy effect. Finally, the potency of TGF-*β* risk score in adjusting precise treatment of HNSC was evaluated.

**Results:**

We systematically established TGF-*β* risk score with multi-level predictive ability. TGF-*β* risk score was employed to predict the tumor microenvironment status, which was negatively associated with NK cells but positively related to macrophages and fibroblasts. It reveals that patients with high TGF-*β* risk score predict “cold” TME status. In addition, higher risk scores indicate higher sensitivity to immunotherapy.

**Conclusion:**

We first construct and validate TGF-*β* characteristics that can predict immune microenvironment phenotypes and immunotherapeutic effect in multiple datasets. Noteworthy, TGF-*β* risk score is helpful for individualized precise treatment of patients with the head and neck squamous cell carcinoma.

## 1. Introduction

The global morbidity and mortality of head and neck squamous cell carcinoma (HNSC) is increasing, which is the sixth most common type of cancer in human beings and causes a great economic burden [1]. Although current treatment strategies are constantly updated, including surgical procedures, immunotherapy, neoadjuvant chemotherapy, radiotherapy, and targeted therapy, surgeons have more options and responses in the treatment of NHSC [2]. Nevertheless, most patients do not respond well to current treatment regimens and cannot effectively obtain the optimal personalized treatment regimens, which leads to high overall mortality [3, 4]. Therefore, it is urgent to investigate new prognostic predictive markers and therapeutic response predictors to promote individualized precise treatment of patients with NHSC. Recent articles have demonstrated that the expression of TGF-*β* pathway regulators is remarkable disordered in head and neck cancer [5, 6]. However, as far as we know, the carcinogenic effect of TGF-*β* pathway on head and neck cancer has not been systematically analyzed, which is worthy of further study.

TGF-*β* pathway modulates tumor pathobiological process, especially in antitumor immune response [7]. TGF-*β* signaling pathway induces apoptosis, inhibits tumor progression, and enhances TME homeostasis [8]. On the other hand, overexpression of autocrine TGF-*β* by transcription factors SNAIL and SLUG stimulates epithelial-mesenchymal transition in tumor tissues, leading to tumor recurrence or drug resistance [9]. TGF-*β* aggrandizes cancer cell immune escape by inhibiting the proliferation, differentiation, and immune ability of various immunocytes (such as dendritic cells, neutrophile cells, and NK cells) [10]. Generally, the activation of TGF-*β* pathway implies low survival rate and leads to resistance to immunotherapy. Although TGF-*β* plays a crucial role in modulating a variety of cancer processes, TGF-*β* related therapy has not been well-explored. With the development of second-generation sequencing and other sequencing methods, more and more genomic characteristics have been explored to portent the clinical outcomes and treatment opportunities of cancer [11, 12]. Zhang et al.elaborated activation of TGF-*β* and WNT signaling pathways may contribute to poor prognosis of HNSC [13]. However, few studies have associated TGF-*β* characteristics with the tumor immune microenvironment phenotype of HNSC.

Nowadays, immuno oncology has attracted much attention due to its special clinical benefits for a variety of cancers. Immunotherapy is a new treatment method that has achieved good results for some cancers, such as colon adenocarcinoma, head and neck squamous cell carcinoma, bladder urothelial carcinoma and breast invasive carcinoma [14–16]. The innovation of immune checkpoint blockers, including PD-1 or PD-L1 monoclonal antibodies has brought hope to patients with advanced HNSC [17]. The patients' clinical response to immunotherapy mainly relies on the tumor microenvironment [18]. Tumor cells, immune cells, and extracellular matrix constitute a whole, called the tumor microenvironment. Types of immune cells and infiltration levels of different cells, such as lymphocytes, neutrophils, and macrophages, are associated with the organism's immune response to tumors [19]. The cell composition (especially immune cells) and matrix components of the tumor microenvironment are regulated by various mechanisms. The most important mechanism is the TGF-*β* signaling pathway. TGF-*β* pathway-related regulators and immune cell infiltration play an indispensable role in tumor development and progression [20]. Therefore, a comprehensive analysis of the relationship between TGF*β* pathway-related regulators and overall survival rate may provide a new reference for the treatment and prognosis of NHSC.

In this study, we employed the single sample gene set enrichment analysis (ssGSEA) algorithm to divide NHSC patients into high and low TGF-beta signaling scores. ssGSEA, which is an extension of the GSEA method, to calculate the enrichment scores of each sample and gene set pair. Unlike the GSEA analysis in group units (such as cancer vs normal), ssGSEA scores are available for each sample. In this way, ssGSEA converts gene expression profiles of a single sample into gene set enrichment profiles. This transformation enables researchers to describe the cell state according to the activity level of biological processes and pathways rather than the expression level of a gene profile. Therefore, if ssGESA uses the gene set related to TGF*beta*pathway-related regulators, the TGF*-beta* pathway score can be calculated. Then the abundance of immune cells in different samples was estimated by TIMER, CIBERSORT, QUANTISEQ, MCP-counter, and EPIC. In addition, the ESTIMATE algorithm was used to calculate the immune score and interstitial score to reflect the microenvironmental state. The *K*-*M* analysis was employed to verify the prediction efficiency of the clinical prognosis and immunotherapy. Considering the complex clinical application of the ssGSEA algorithm, we used univariate Cox, LASSO, and multivariate COX regression analysis to determine the genetic characteristics of five prognostic-relatedTGF-beta signaling pathways. Finally, we assessed the accuracy of prognostic features of TGF-beta signaling pathway related genes. The potential mechanism of TGF-beta signaling pathway related gene features can not only enhance the ability to predict the prognosis of NHSC patients but also explain the potential mechanism.

## 2. Method

### 2.1. Data Preprocessing

This study included eight independent HNSC queues, of which only the TCGA-HNSC queue was listed as an RNA-seq dataset and downloaded from the USCS Xena website. In addition, the seven HNSC queues are microarray datasets and downloaded from E_MTAB or GEO databases, including GSE41613, GSE42743, GSE65858, GSE75538, GSE8471, GSE117973, and E_MTAB_8588. At the same time, matching clinical data were included. After eliminating the samples with repeated sequencing or with clinical data loss, 1356 HNSC samples were eventually included for ssGSEA. Then, in order to obtain each patient's absolute TGF-beta signalling pathway enrichment, a total of 233 TGF-signaling pathway regulators were obtained from the previous literature and packaged as TGF. Gmt.

### 2.2. Identification of TGF-Beta Signaling Pathway-derived Score

In this research, the LASSO-Cox regression analysis was employed to construct the predicted model. The GSE65858 dataset was used as the training set, and the GSE41613 dataset was used as the external validation set. Before modeling, the combat function in the sva package was used to remove the batch effect of the GSE65858 and GSE41613 datasets. Firstly, univariate cox regression analysis was performed on 233 TGF-*β* signaling pathway regulatory factors in GSE65858 cohort to identify prognostic-related genes. Subsequently, the Least Absolute Shrinkage and Selection Operator (LASSO) model was used to remove redundant genes, and the risk-score formula was established by multivariate Cox regression analysis of the integration coefficient and gene expression value. According to the median value of the risk score formula, patients were divided into high-risk and low-risk subtypes. In addition, based on the encapsulated TGF. Gmt file, the TGF-beta signaling pathway absolute enrichment of each patient in GSE41613, GSE42743, GSE65858, GSE75538, GSE8471, GSE117973, and E_MTAB_8588 was calculated using ssGSEA algorism, and patients were divided into high and low risk subgroups using the median of TGF-beta signaling pathway-derived score in each cohort as cut-off value. The time-dependent receiver operating characteristic curve was employed to calculate the prediction performance of TGF-*β* risk score.

### 2.3. Enrichment Analysis

The Gene ontology (GO) algorithm was performed to commentate on the physiopathological processes, including biological processes, molecular functions, and cellular components. The Kyoto Encyclopedia of Genes and Genomes (KEGG) commentates on physiopathological pathways. Limma packs were used for differential analysis of high-score and low-score samples from eight different cohorts, and the differential genes were overlapped, followed by enrichment analysis of overlapped genes. *P* value <0.05 and *q* value <0.05 were considered to be a significant enrichment pathway.

### 2.4. Immunological Analysis and Immunotherapy Cohort

Considering that a variety of immune cells are required to participate in the antitumor immunity process of HNSC, different algorithms, such as TIMER, CIBERSORT, QUANTISEQ, McP-counter, and EPIC, were used to estimate the componence of immunocytes in different samples for immune cell analysis. In addition, the ESTIMATE algorithm was used to calculate the immune score and interstitial score to reflect microenvironmental status. It is worth noting that the E_MTAB_8588 dataset cannot be calculated by some algorithms due to its different expression spectrum format (*Z*-score) from the other seven datasets and lack of original data for conversion. Therefore, we ignore this dataset in the immune cell score. In addition, Spearman correlation analysis was performed for TGF-beta signaling pathway-derived score and mRNA expression of immune modulators. Considering the strong indication of TGF-beta for immunotherapy, GSE111636, GSE91061, GSE126044, GSE78220, GSE136961, GSE35640, GSE173839, and GSE100797 TGF-beta scores were performed in the GSE115821 immunotherapy cohort, and survival and immunotherapy response were assessed.

### 2.5. Statistical Analysis

R software (version 4.0.5) was used for data visualization and statistical analysis. The Pearson or Spearman correlation analysis was used to study the relationship and strength between variables. The ROC curve was plotted to evaluate the predictive performance of the TGF-*β* risk score in predicting prognosis. Analyses with *P* < 0.05 on both sides were considered statistically significant.

## 3. Results

### 3.1. TGF-Beta Signaling Score Based on ssGSEA in the Multicenter Study

**Figure 1:**
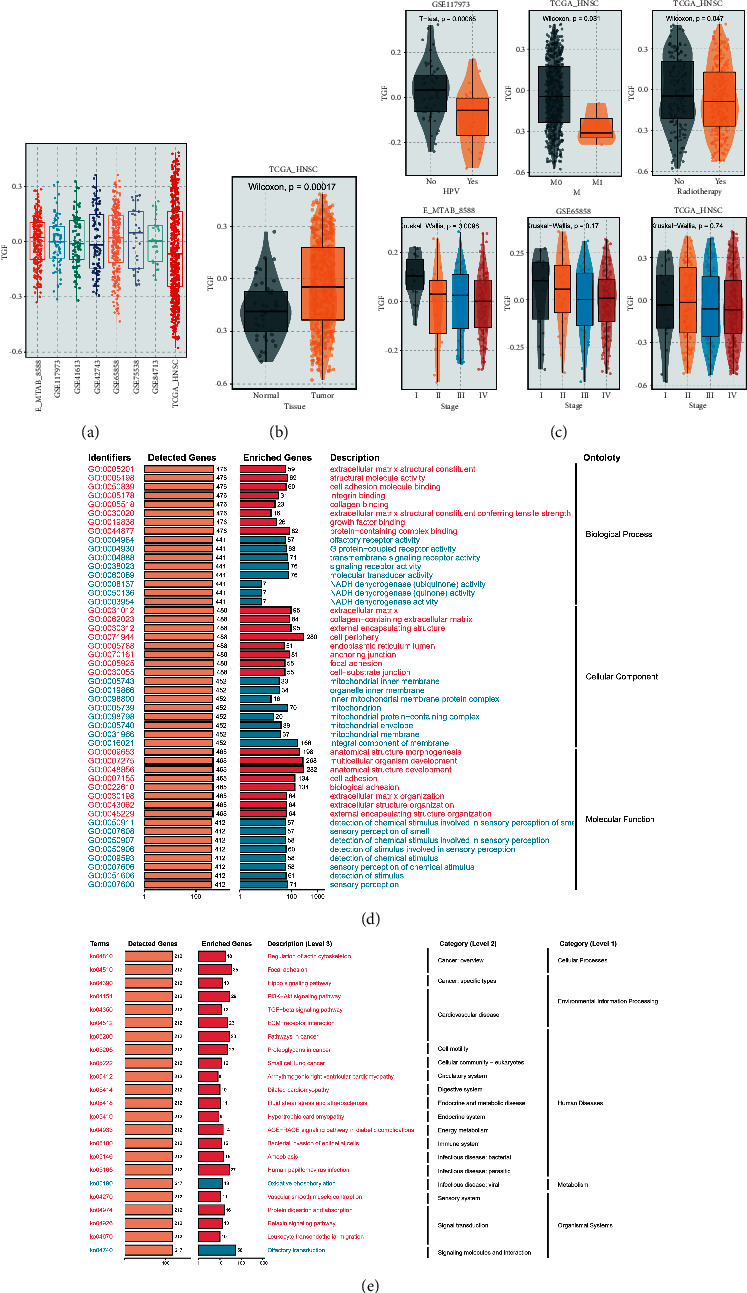
Construction of TGF-*β* signal score in eight datasets. (a) The scatter diagram indicated the distribution of TGF-*β* signal score across eight datasets. (b) The scatter diagram exhibited the TGF-*β* signal score in HNSC and normal samples. (c) Relationship between TGF-*β* signal score, HPV infection status, *M* stage and clinical stage in TCGA_HNSC and GEO database. (d) The top 8 results of GO analysis (BP, CC, and MF) in TGF-*β* signal pathway_H (red) and TGF-*β* signal pathway_L (blue). (e) The results of KEGG analysis in TGF-*β* signal pathway_H (red) and TGF-*β* signal pathway_L (blue).

Figure 1(a) shows the TGF- beta signaling pathway absolute enrichment score in each dataset. We analyzed the correlation between TGF- *β* signaling pathway absolute enrichment score and tumor STATUS. In the TCGA-HNSC dataset, the TGF-*β* risk score of tumor samples was higher than that of normal tissues (Figure 1(b)). Interestingly, we found that tumor samples with distant metastasis had lower risk scores, and risk scores were associated with clinical stage in the E-MTAB-8588 and GSE65858 and TCGA-HNSC datasets, with higher stage tumor samples owning lower TGF-*β* risk scores. Then we found that HPV-positive patients had lower risk scores (Figure 1(c)).

The limma packages were used to perform differential analysis of high-score and low-score samples from 8 different cohorts, and the differential genes were overlapped. For GO_Biological Process analysis, these TGF-signaling pathway regulator related genes were found to be enriched in a variety of pathways, including cell adhesion, integrin binding, and collagen binding, which were all positively correlated, and G protein-coupled receptor activity, which was negatively correlated.For GO_Cellular Component analysis, these TGF-*β* signaling pathway modulator related genes regulated focal adhesion, cell periphery. For GO_Molecular Function analysis, TGF-*β* signaling pathway modulator related genes was related to cell adhesion (Figure 1(d)). The GO analysis may summarize that the TGF-*β* signaling pathway affects the tumor cells metastasis. In addition, KEGG analysis showed that TGF-*β* signaling modulator related genes were also abundant in the TGF-*β* signaling pathway, hippo signaling pathway, and PI3K-Akt signaling pathway, and pathways in cancer (Figure 1(e)). As expected, the TGF-*β* signaling pathway was clearly the most common enrichment pathway for these DEGs.

### 3.2. TGF-Beta Signaling Score is Associated with Prognosis in HNSCC Patients

**Figure 2:**
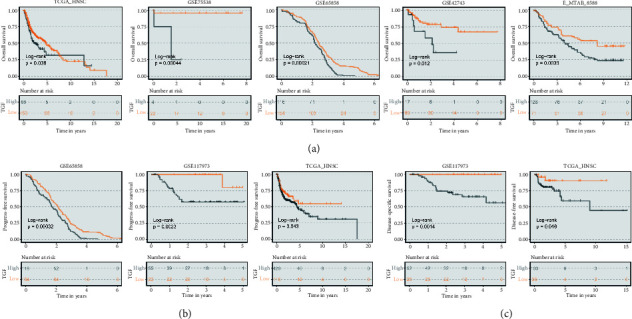
The prognostic value of TGF-*β* signal score. (a) Patients in the low-TGF-*β* signal score group (orange) displayed better overall survival than those in the high- TGF-*β* signal score group (gray). (b) Patients in the low-TGF-*β* signal score group (orange) displayed better progress-free survival than those in the high- TGF-*β* signal score group (gray). (c) Patients in the low-TGF-*β* signal score group (orange) displayed better disease-free survival than those in the high-TGF-*β* signal score group (gray).

Patients in different cohorts were divided into high TGF- risk groups and low TGF- risk groups based on the optimal cutoff value of the TGF- risk score.K-M curve showed that the overall survival rate of patients with high risk was worse, and the overall survival rate of patients with low risk was better, which was verified in TCGA-HNSC, GSE75538, GSE65858, GSE42743 and E_MTAB_8588 datasets (Figure 2(a)). In addition, this risk score can also be used to predict progression-free survival time and disease-specific survival time. The high-score group had a poor prognosis, while the low-score group had the opposite prognosis (Figures 2(b) and 2(c)).

### 3.3. TGF-Beta Signaling Score Represents Different Immune Cell Infiltration

**Figure 3:**
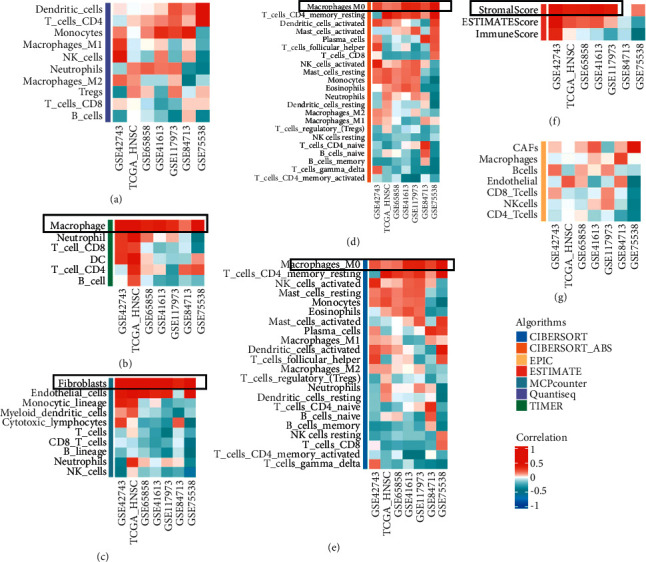
The relationship between TGF-*β* signal score and immunocytes infiltration in seven databases. The heat map displayed the relationship between TGF-*β* signal score and the level of immunocytes infiltration in tumor microenvironment using Quantiseq (a), TIMER (b), MCPcounter (c), CIBERSORT_ABS (d), CIBERSORT (e), ESTIMATE (f), EPIC (g), algorism.

The immune infiltration status of patients affects the fate of tumor cells and predicts the sensitivity of patients to immunotherapy. Firstly, we used seven different algorithms to describe the immune cell infiltration status or immune score level in the tumor immune microenvironment of each patient in each dataset.  Figure 3(a) shows the results of Quantiseq algorithm, Figure 3(b) shows the results of TIMER algorithm, Figure 3(c) shows the results of MCPcount algorithm, Figure 3(d) shows the results of CIBERSORT_ABS algorithm, Figure 3(e) shows the results of CIBERSORT algorithm, Figure 3(f) shows the results of ESTIMATE algorithm, and Figure 3(g) shows the results of EPIC algorithm. We found that TGF-beta signaling score was positively correlated with macrophages and fibroblasts, but negatively correlated with NK cells. Therefore, we speculate that the immune microenvironment of patients with a high-risk score has tumor-promoting activity, and a low-risk score predicts that the tumor microenvironment is in a hot TME state.

### 3.4. TGF-Beta Signaling Score Associated with Immunomodulator Expression

**Figure 4:**
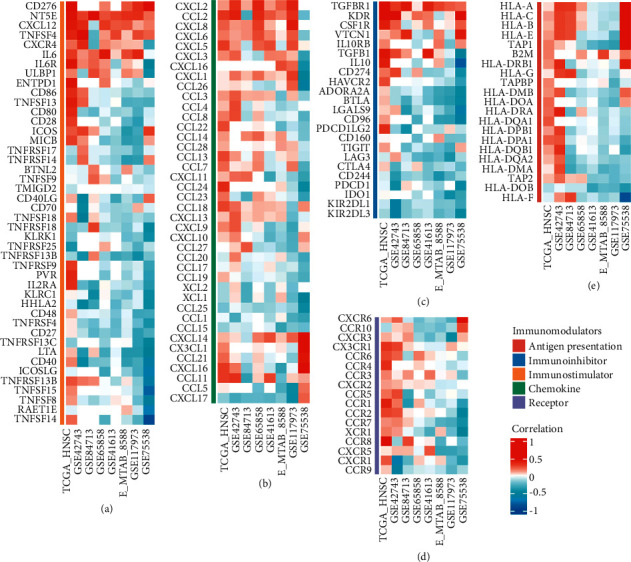
The relationship between TGF-*β* signal score and immunomodulators in seven databases. The heat map displayed the relationship between TGF-*β* signal score and the level of immunomodulators. Immunostimulator (a), chemokine (b), immunoinhibitor (c), receptor (d), antigen presentation (e), respectively.

In addition, we collected antigen presentation, immunoinhibitor, immunostimulator, chemokine, and receptor-related factors to explore the correlation between TGF-beta signaling score and immune regulators in each patient.  Figure 4(a) displayed the predictive result of the correlation between TGF-beta signaling score and immunostimulator.  Figure 4(b) shows the prediction results of the correlation between TGF-beta signaling score and cytokines, and Figure 4(c) shows the prediction results of the correlation between TGF-beta signaling score and immunoinhibitors.  Figure 4(d) shows the prediction results of the correlation between TGF-beta signaling score and receptors, and Figure 4(e) shows the prediction results of the correlation between TGF-beta signaling score and antigen presentation. We found that TGF-beta signaling score was positively correlated with immunomodulators such as CD276, NT5E, CXCL12, TNFSF4, CXCL2, CCL2, CXCL8, TGFBR1, and KDR, and significantly negatively correlated with CXCL17, KIR2DL3, and KIR2DL1.

### 3.5. TGF-Beta Signaling Score Strongly Reflects Immunotherapy Response

**Figure 5:**
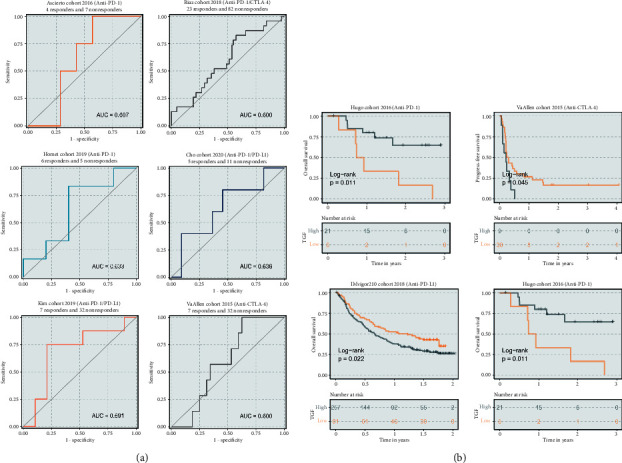
The TGF-*β* signal score promoted individual immunotherapy for HNSC. (a) ROC curve of TGF-*β* signal score in Ascierto cohort 2016, Riaz cohort 2018, Homet cohort 2019, Cho cohort 2020, Kim cohort 2019, and VanAllen cohort 2015, respectively. (b) Patients in the low-TGF-*β* signal score group (orange) displayed better overall survival than those in the high- TGF-*β* signal score group (gray) when using CTLA-4 and PD-L1 monoclonal antibody, while patients in the low-TGF-*β* signal score group (orange) displayed worse overall survival than those in the high- TGF-*β* signal score group (gray) when using PD-1 monoclonal antibody.

Considering the strong indication of TGF-beta on immunotherapy, we calculated the TGF-beta score in the immunotherapy cohort and evaluated the clinical outcomes, and immune response.  Figure 5(a) is the ROC curve of each dataset. The area under the curve is greater than 0.6, indicating that the model has good prediction performance.  Figure 5(b) displays that patients with high-risk score have a good therapeutic effect on PD-1 monoclonal antibody and a poor therapeutic effect on CTLA-4 and PD-L1 monoclonal antibody, which means that patients with a high-risk score are more suitable for PD-1 monoclonal antibody treatment.

### 3.6. TGF-Beta Signaling Score-derived Risk Score (Lasso-Cox) can be Better used in Clinics

**Figure 6:**
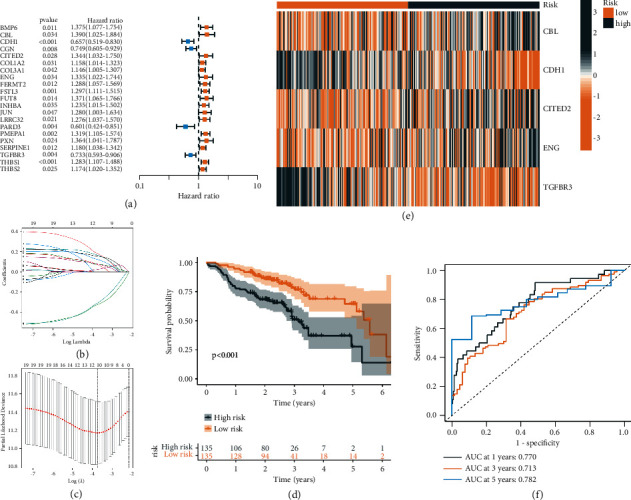
Development of a TGF- *β* signaling score-derived risk score (lasso-cox). (a) TGF-*β* signal related prognostic genes were presented by dendrogram. (b) LASSO coefficients of 10 prognostic TGF-*β* genes. (c) Cross-validation for turning parameter selection via minimum criteria in the LASSO regression model. (d) Kaplan—Meier analysis of OS for TGF-*β* risk score. (e) Heat map displayed the expression of 5 prognostic genes in HNSC and normal samples. (f) ROC curves of the TGF-*β* risk score in predicting OS.

Thus, TGF-beta signaling pathway regulators can be used to effectively predict the prognosis of patients with the head and neck squamous cell carcinoma and the clinical response to immunotherapy. However, considering the complexity of clinical application of the ssGSEA algorithm, the Lasso-cox algorithm had stronger clinical applicability. We used expression × coefficient to calculate TGF-beta signaling score. To begin, univariate Cox regression analysis was used to screen 21 prognostic-related regulatory factors of the TGF-beta signalling pathway (Figure 6(a)). The Lasso regression model reduced the dimension of candidate prognostic genes, with a total of 10 prognostic genes included in the model (Figures 6(b) and 6(c)). Finally, a multivariate COX regression model was used to construct a risk model.

(1) $$\begin{array}{c} \text{Risk score}=\left(0.3850*\text{CBL}\right)+\left(-0.4569*\text{CDH}1\right)+\left(0.3703*\text{CITED}2\right)+\left(0.3106*\text{ENG}\right)+\left(-0.4749*\text{TGFBR}3\right). \end{array}$$

**Figure 7:**
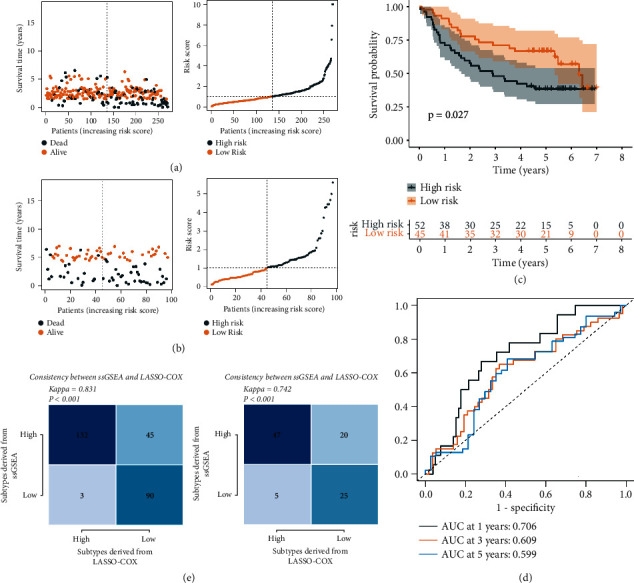
External validation of the TGF-*β* risk score. (a)-(b) The scatter plot revealed survival rates in the high- and low- TGF-*β* risk score groups in GEO dataset. (c) Kaplan–Meier analysis of OS for TGF-*β* risk score. (d) ROC curves of the TGF-*β* risk score in predicting OS. (e) The Kappa coefficient revealed the consistency of the ssGSEA algorism and lasso-cox algorithm.

According to the risk median, the patients were divided into high and low risk groups. The *K*-*M* curve showed that the patients in the high-risk group showed poor clinical results, and the patients in the low-risk group showed longer survival times (*P* value <0.001, Figure 6(d)). The heatmap showed that CBL, CITED2, and ENG were highly expressed in the high-risk group, while CDH1 and TGFBR3 were lowly expressed (Figure 6(e)). ROC curve demonstrated that the 1-year survival rate prediction performance was 0.770, the 3-year prediction rate was 0.713, and the 5-year prediction rate was 0.782 (Figure 6(f)). In addition, we conduct verification in the external dataset, and the results are consistent with the above (Figures 7(a)–7(c), *P* value = 0.027), ROC curve displayed that the 1-year survival rate prediction performance was 0.706, the 3-year prediction rate was 0.609, and the 5-year prediction rate was 0.599 (Figure 7(d)). The consistency test found that the kappa value was greater than 0.7, suggesting that it was reliable to replace the ssGSEA algorithm with LASSO-COX (Figure 7(e)).

## 4. Discussion

Previous literature has reported that the TGF-*β* pathway is a key factor in tumorigenesis. However, TGF-*β* has two “tracks.” From one perspective, TGF-*β* protects tumor cells from malignant evolution [21]. In turn, TGF-*β* regulation processes, such as proliferation, differentiation, and immunosuppression, may be used by cancer cells [22]. In addition, TGF-*β* expression and activation can be considered as a potential target for antitumor therapy [23]. With the discovery of the biological mechanism of TGF-*β* regulating tumor, the therapeutic ability of TGF-*β* has been paid more and more attention.

At present, TGF-*β* signaling pathway is widely utilized to predict the prognosis of various tumors, such as bladder cancer, hepatocellular carcinoma, and breast cancer [24–26]. Previous work has shown that TGF-*β* is essential for the tumorigenesis and invasion of head and neck squamous cell carcinoma. For example, TGF-*β* promotes tumor metastasis by regulating lncRNA, EPB41L4A-AS2 [27]. Wang et al. demonstrated that up-regulated TGF-*β* promotes epithelial-mesenchymal transition (EMT) through STAT3 activation, which drives invasion and metastasis of head and neck squamous cell carcinoma [28]. In addition, Zheng et al. established seven TGF-*β*-pathway related gene features with good prediction efficiency. The model was employed to predict the efficacy of immunotherapy and chemotherapy such as cisplatin, erlotinib, paclitaxel, and crizotinib [29]. These studies have shown that TGF-*β* is closely related to head and neck squamous cell carcinoma. However, few studies systematically explore the role of TGF-*β*-related characteristics in regulating TME characteristics and predicting the prognosis of HNSC. Therefore, we prioritized the role of TGF-*β* in HNSC in this article to make up for this gap. We have extracted TGF-*β* related gene sets from previously published literature. Then, according to our algorithm, we selected five important genes to construct TGF-*β* characteristics. These genes include CBL, CDH1, CITED2, ENG, and TGFBR3.

These five genes are related to tumor progression. Belizaire et al. demonstrated that increased LYN activation and interaction with mutant CBL promoted CBL phosphorylation, phosphoinositol 3-kinase regulatory subunit 1 (PIK3R1) recruitment and downstream phosphatidylinositol 3-kinase (PI3K)/AKT signal transduction, thereby promoting tumor progression [30]. Zhuang et al. found that miR-204-5p inhibited epithelial-mesenchymal transition (EMT) and STAT3 signal transduction by targeting SNAI2, SUZ12, HDAC1, and JAK2. In addition, the above factors formed inhibitory complexes on the CDH1 promoter to maintain the EMT state of tumor tissue. CDH1 modulates the status of head and neck squamous cell carcinoma, according to these studies [31]. Shin et al. demonstrated that CITED2, as a molecular chaperone, leads PRMT5 and p300 to nucleolin, thereby activating nucleolin and promoting metastasis in cancer [32]. TGF-*β* type III receptor (TGFBR3) is an important part of TGF-*β* signal transduction, which acts as tumor suppressor in tumor and stromal cells through SMAD4-dependent and non-dependent manner during head and neck squamous cell carcinogenesis. Based on these five genes, we organized multiple HNSC datasets (TCGA-NHSC, GSE41613, GSE42743, GSE65858, GSE75538, GSE8471, GSE117973, and E_MTAB_8588) to explore and verify high-value TGF-*β* risk score.

TGF-*β* signal transduction has been proved to inhibit the main factors of adaptability and innate immune response in the progression of tumor [33]. We evaluate the characteristics of TME from a TGF-*β* signal related risk score perspective. First, we verified that TGF-*β* risk score predicted clinical outcomes and immune characteristics in tumor microenvironment. As shown in our figure, patients with higher risk scores always have a lower OS rate, higher tumor grading, and staging. Secondly, we screened the TGF-*β* signal related genes between high-risk and low-risk scoring groups. The characteristics of cell adhesion, integrin binding, and collagen binding in patients with a high-risk score were significantly enriched. Immunotherapy significantly increased the survival rate of patients with advanced NHSC. Therefore, it is urgent to develop accurate immunotherapy indicators. Here, we associate the risk score with the expression of immunoregulatory factors, including antigen presentation, immunoinhibitor, immunostimulator, chemokine, and receptor-related factors. The results showed that patients with a lower risk score had more therapeutic effects on PD-L1 and CTLA-4 monoclonal antibodies but had poor therapeutic effects on PD-1 monoclonal antibodies, indicating that patients with a lower risk score were not suitable for PD-1 monoclonal antibody treatment. In addition, this study was based on the TCGA and GEO public databases, which require our data set to confirm. However, there are still deficiencies and doubts. The results also showed that the risk score was positively related to macrophages, which was regarded as a semaphore to prevent anti-cancer immunity. Therefore, the pre-existinganti-cancer activity of high-risk score patients may be limited by high levels of tumor-infiltrating macrophages (*M*2) and overexpression of many inhibitory immune checkpoints such as TGFBR1 and KDR.

Korkut et al. proposed a detailed analysis of genomic changes in TGF-*β* signaling pathways in pan-cancer. Correlation analysis showed that TGF-*β* signal mutation, amplification, deletion, DNA methylation, and miRNA changes were found in each type of cancer, and the mutation hot spot of TGF-*β* was determined, indicating that potential biomarkers can be used for further treatment and cancer diagnosis. Their work provides a broad molecular perspective for future research on the function and treatment of multiple cancer pathways mediated by TGF-*β* superfamily. We compare our work with published research. First of all, our candidate genes were screened from TGF-*β* related gene sets, using Lasso algorithm and COX regression model, respectively. Secondly, we constructed a risk model consisting of five genetic characteristics and associated it with tumor microenvironment immune characteristics and individualized treatment strategies. Third, we employed multiple GEO queues to verify the predictive performance of our model. Therefore, our model may predict the prognosis of HNSC and guide individualized precision medicine [34–36]. It is worth noting that this topic accurately screens the key genes, constructs the prognosis model efficiently, associates the model with immune profiles, and evaluates the predictive performance of clinical outcomes and patients' clinical responses to immunotherapy. Expected results were achieved in the data analysis.

However, several aspects of our research inevitably have defects. First of all, all our results are based on public data. Due to the limitation of our clinical data, more prospective data are urgently needed to further verify the characteristics of TGF-*β*. Second, the clinical application of TGF-*β* features needs more exploration. The risk model is based on RNAseq data, not combined with time, space, and single cell RNA transcriptome data. In addition, we will design the corresponding gene detection kit according to the five genes of the risk model. Thirdly, we dogmatically regard the median TGF-*β* risk score as the critical value of all analyses, which needs further verification. The lack of prospective validation of ICB treatment datasets in HNSC cohorts makes it difficult to further explore the association between immunotherapy and risk score. In the future, multicenter, large-sample immunotherapy cohort investigations will be conducted to further examine the potential of this model to predict clinical treatment of immunotherapy. In conclusion, we have made a small step forward in exploring the reasonable prognostic indicators of HNSC. We developed and verified a well-founded TGF-*β* risk score based on real clinical data. The TGF-*β* risk score has been proved to be able to stratify the clinical results and TME phenotypes of HNSC. It also verified the sensitivity of HNSC immunotherapy.

## 5. Conclusion

Our study provided an indispensable reference for further investigation of the role of TGF-*β* in the tumor microenvironment and immunotherapy efficiency, and rendered personalized prognosis monitoring and potential biological treatment targets for HNSC.

## Data Availability

All datasets generated for this study are included in the article material, including TCGA database (https://portal.gdc.cancer.gov/), and GEO dataset (https://www.ncbi.nlm.nih.gov/gds/): GSE41613, GSE42743, GSE65858, GSE75538, GSE8471, GSE117973, and E_MTAB_8588 (https://www.ebi.ac.uk/arrayexpress/experiments/E-MTAB-8588/).
